# Hydnaceous fungi of China 8. Morphological and molecular identification of three new species of *Sarcodon* and a new record from southwest China

**DOI:** 10.3897/mycokeys.66.49910

**Published:** 2020-04-03

**Authors:** Yan-Hong Mu, Ya-Ping Hu, Yu-Lian Wei, Hai-Sheng Yuan

**Affiliations:** 1 CAS Key Laboratory of Forest Ecology and Management, Institute of Applied Ecology, Chinese Academy of Sciences, Shenyang 110164, China Institute of Applied Ecology, Chinese Academy of Sciences Shenyang China; 2 University of the Chinese Academy of Sciences Beijing China; 3 University of the Chinese Academy of Sciences, Beijing 100049, China Institute of Environmental Sciences, Ministry of Ecology and Environment Nanjing China; 4 Research Center for Nature Conservation and Biodiversity of Nanjing Institute of Environmental Sciences, Ministry of Ecology and Environment/ State Environmental Protection Scientific Observation and Research Station for Ecological Environment of Wuyi Mountains, Nanjing 210042, China State Environmental Protection Scientific Observation and Research Station for Ecological Environment of Wuyi Mountains Nanjing China

**Keywords:** Bankeraceae, ITS and LSU, new species and record, taxonomy, Thelephorales

## Abstract

Three new stipitate hydnoid fungi, *Sarcodon coactus*, *S. grosselepidotus* and *S. lidongensis*, are described and illustrated, based on morphological characteristics and nuc ITS rDNA + nuc LSU rDNA sequence analyses and a new record, *S. leucopus*, from China is reported. *S. coactus* is characterised by ellipsoid to round basidiocarps, reddish-brown to dark brown, felted pileal surface with white and incurved margins, simple-septate and partly short-celled generative hyphae and irregular subglobose, thin-walled, brown basidiospores with tuberculate ornamentation (tuberculi up to 1 μm long). *S. grosselepidotus* is characterised by infundibuliform to round, occasionally deeply fissured pileus, pale orange to dark ruby pileal surface with ascending and coarse scales, simple-septate generative hyphae and irregular ellipsoid to globose, thin-walled, brown basidiospores with tuberculate ornamentation (tuberculi up to 0.7 μm long). *S. lidongensis* is characterised by plano-convex to somewhat depressed and regular orbicular pileus, light brown to dark brown pileal surface with adhering squamose and purplish-brown, incurved and occasionally incised margin, cylindrical or broadened below stipe, simple-septate generative hyphae and irregular ellipsoid to subglobose, thin-walled basidiospores with tuberculate ornamentation (tuberculi up to 1 μm long). The absence of the clamp connection is the common morphological characteristic of these three new species; however, *S. leucopus*, a new record from China, has frequently clamped generative hyphae. Molecular analyses confirm the phylogenetic positions of three new and the new record species. The discriminating characters of these three new species and closely related species are discussed and a key to the species of *Sarcodon* from China is provided.

## Introduction

The genus *Sarcodon* Quél. ex P. Karst. (1881), together with *Bankera* Coker and Beers ex Pouzar (1955), *Hydnellum* P. Karst. (1896) and *Phellodon* P. Karst. (1881), belong to Bankeraceae, Thelephorales of Basidiomycota. They are a group of stipitate hydnoid fungi that inhabit the soil (Maas Geesteranus 1975).

Species of Bankeraceae are ectomycorrhizal fungi which associate with many kinds of angiosperm and gymnosperm trees, especially with Pinaceae and Fagaceae, such as *Pinus strobus*, *Picea sitchensis*, *Fagus grandifolia*, *Quercus rubra* and *Castanea sativa* (Maas Geesteranus 1975; Harrison 1984; Baird 1986; Baird et al. 2013) and usually occur in natural and comparatively undisturbed forests (Arnolds 1989). They can obtain energy from and transport nutrients to the host plants and are of great ecological significance in promoting forest vegetation recovery (Gardes and Bruns 1996; Erland and Taylor 1999). These fungi are vulnerable to impact due to changes in the environment, such as habitat loss, nitrogen deposition, decrease of host tree species and subsequently increased ground temperatures (Arnolds 1989; Otto 1992; Vesterholt et al. 2000; Newton et al. 2002; Arnolds 2010; Baird et al. 2013). In Europe, stipitate hydnoid fungi have been considered one of the most endangered groups of macrofungi and have been included in Red Data Lists (Hrouda 1999; Walleyn and Verbeken 2000; Hrouda 2005; Nitare 2006; Senn-Irlet et al. 2007), which have been used as indicators that forests need to be protected (Ainsworth 2005; Nitare 2019).

The genus *Sarcodon* is characterised by solitary to gregarious, stipitate, pileate basidiocarps, hydnaceous hymenophore, the monomitic hyphal system owning inflating or not inflating hyphae, the presence or absence of clamp connections and irregular ellipsoid to globose, tuberculate basidiospores which are brown in mass. Besides, the dry basidiocarps often produce farinaceous to fragrant or acidic odour (Maas Geesteranus 1971; Baird 1986; Arnolds 2003; Baird et al. 2013). In morphology, *Sarcodon* is closely related to *Hydnellum*, but the former usually has soft and fleshy basidiocarps and the latter has hard and corky basidiocarps (Maas Geesteranus 1971; Larsson et al. 2019). The macro-morphology of these two genera often depends on their environmental parameters, such as precipitation, temperature or obstructions. Additionally, the variable growth of basidiocarps makes it difficult to distinguish each other. Therefore, it is essential to support and confirm their identities using molecular sequence data (Baird et al. 2013). Recent molecular phylogenetic analyses reveal that *Sarcodon* and *Hydnellum* form paraphyletic lineage and suggest using the spore length as the delimitation between the two genera. *Hydnellum* species had spore lengths in the range 4.45−6.95 µm, while the corresponding range for *Sarcodon* was 7.4−9 µm (Larsson et al. 2019).

Most species of *Sarcodon* have been reported from the northern temperate hemisphere (Maas Geesteranus 1971, 1975; Baird 1986; Stalpers 1993; Pegler et al. 1997; Dai 2011) and are commonly found in North America (Harrison 1964, 1984; Baird 1985, 1986; Baird et al. 2013), Netherlands (Maas Geesteranus 1956, 1976), Spain (Pérez-De-Gregorio et al. 2011), France (Pieri and Rivoire 1994), Russia (Baird 1985) and Italy (Caclalli and Caroti 2005). Some species have also been recorded from southern hemisphere, such as New Zealand (Maas Geesteranus 1964, 1971, 1975) and Australia (Mleczko et al. 2011; Magnago et al. 2015; Hahn et al. 2018). Around 59 species have been described or transferred to the genus according to Index Fungorum (http://www.indexfungorum.org/) and MycoBank, but only five taxa have been reported from China (Dai 2011). In addition, some species of *Sarcodon* have medicinal functions, for instance, lowering cholesterol, antioxidant, antibacterial, anti-tumour, improving immunity etc. (Wu et al. 2019).

Investigations of hydnaceous fungi in China have been carried out in recent decades and many *Sarcodon* specimens have been collected. During the study of these specimens, three undescribed species and a new record species have been identified using morphological characters and phylogenetic analyses of nuc rDNA ITS1-5.8S-ITS2 combined with nuc 28S rDNA sequences. Here, we describe them in this paper.

## Materials and methods

### Morphological studies

Specimens are deposited at the herbarium of the Institute of Applied Ecology, Chinese Academy of Sciences (IFP). Microscopic procedures follow Mu et al. (2019). Microscopic studies used sections mounted in Cotton Blue (CB): 0.1 mg aniline blue dissolved in 60 g pure lactic acid; CB− = acyanophilous. Amyloid and dextrinoid reactions were tested in Melzer’s reagent (IKI): 1.5 g KI (potassium iodide), 0.5 g I (crystalline iodine), 22 g chloral hydrate, 20 ml distilled water; IKI− = neither amyloid nor dextrinoid reaction. Sections were mounted in 5% KOH (potassium hydroxide) and studied at magnifications up to 1000× using a Nikon Eclipse E600 microscope (Tokyo, Japan) with phase contrast illumination. Dimensions were estimated subjectively with an accuracy of 0.1 μm. In presenting basidiospore size ranges, 5% of the measurements at each end of the range are given in parentheses. The following abbreviations are used in the text: L_m_ = mean spore length, W_m_ = mean spore width, Q = range of length/width ratios for specimens studied and n = total number of basidiospores measured from a given number of specimens. The surface morphology for the basidiospores was observed with a Phenom Prox scanning electron microscope (ESEM, Phenom Prox, FEI, The Netherlands) at an accelerating voltage of 10 kV. A thin layer of gold was coated on the samples to avoid charging. Special colour terms are from Rayner (1970) and Munsell (2015).

### Molecular procedures and phylogenetic analyses

Fungal taxa and strains used in this study are listed in Table 1. Phire Plant Direct PCR Kit (Thermo Fisher Scientific) procedures were used to extract total genomic DNA from the basidiocarps. Polymerase chain reactions (PCR) were performed on a Bio-Rad T100 Thermal cycler (Bio-RAD Inc). Amplification reactions were performed in a 30 μl reaction mixture using the following final concentrations or total amounts: 0.9 μl template DNA, 15 μl of 2× Phire Plant PCR buffer, 1.5 μl of each primer, 0.6 μl Phire HS II DNA Polymerase and 10.5 μl ddH_2_O (double distilled water). The nuc rDNA ITS1-5.8S-ITS2 region (ITS) was amplified with the primers ITS1-F (5' CTTGGTCATTTAGAGGAAGTAA 3') and ITS4 (5' TCCTCCGCTTATTGATATGC 3') (Baird et al. 2013; Loizides et al. 2016). The 28S nuclear rDNA region was amplified with the primers LROR (5' ACCCGCTGAACTTAAGC 3') and LR7 (5' TACTACCACCAAGATCT 3') (Vizzini et al. 2016). The PCR thermal cycling programme conditions were set as follows: initial denaturation at 98 °C for 5 min, followed by 39 cycles at 98 °C for 30 s, × °C (the annealing temperatures for ITS1-F/ITS4 and LROR/LR7 were 57.2 °C and 47.2 °C, respectively) for 30 s, 72 °C for 30 s and a final extension at 72 °C for 1 min. PCR amplification was confirmed on 1% agarose electrophoresis gels stained with ethidium bromide (Stöger et al. 2006). DNA sequencing was performed at the Beijing Genomics Institute (BGI). All newly generated sequences were submitted to GenBank. Additional ITS rDNA and LSU rDNA sequences in the dataset, used to establish phylogenetic relationships, were downloaded from GenBank (http://www.ncbi.nlm.nih.gov/genbank) and UNITE (https://unite.ut.ee/index.php) (Table 1).

**Table 1.** Voucher numbers, geographic origins and GenBank accession numbers for the specimens and included, in boldface, are sequences produced in this study.

Species	Geographic origin	Voucher number	GenBank Accessions	
			ITS	28S
*Amaurodon aquicoeruleu* Agerer	Australia	Isotype in M	AM490944	AM490944
*Hydnellum aurantiacum* (Batsch) P. Karst.	Norway	OF29502	MK602713	MK602713
*H. aurantiacum*	Norway	EBendiksen177-07	MK602712	MK602712
*H. auratile* (Britzelm.) Maas Geest.	Norway	OF242763	MK602715	MK602715
	Norway	OF294095	MK602714	MK602714
*H. caeruleum* (Hornem.) P. Karst.	Norway	EBendiksen584-11	MK602719	MK602719
	Norway	EBendiksen575-11	MK602718	MK602718
*H. complicatum* Banker	USA	REB-329	KC571712	
	USA	REB-71	KC571711	
*H. concrescens* (Pers.) Banker	Norway	O-F-251488	UDB036247	
*H. cristatum* (Bres.) Stalpers	USA	REB-88	KC571718	
	USA	REB-169	JN135174	
*H. cumulatum* K.A. Harrison	Finland	TU115384	UDB011871	UDB011871
	Estonia	TU111191	UDB032402	
*H. cyanopodium* K.A. Harrison	USA	SEW 85	AY569027	
*H. diabolus* Banker	Canada	KAH13873	AF351863	
*H. dianthifolium* Loizides	Italy	ML902162HY	KX619420	
	Cyprus	ML61211HY	KX619419	
*H. earlianum* Banker	USA	REB-75	KC571724	
	USA	REB-375	JN135179	
*H. ferrugineum* (Fr.) P. Karst.	Sweden	ELarsson197-14	MK602722	MK602722
	Norway	OF297319	MK602720	MK602720
*H. ferrugipes* Coker	USA	REB-176	KC571727	
	USA	REB-324	KC571728	
*H. geogenium* (Fr.) Banker	Norway	OF66379	MK602723	MK602723
	Norway	OF296213	MK602724	MK602724
*H. gracilipes* (P. Karst.) P. Karst.	Sweden	GB-0113779	MK602727	MK602727
	Sweden	ELarsson219-11	MK602726	MK602726
*H. mirabile* (Fr.) P. Karst.	Sweden	SLund140912	MK602730	MK602730
	Sweden	ELarsson170-14	MK602729	MK602729
*H. peckii* Banker	Norway	EBendiksen567-11	MK602733	MK602733
	Sweden	ELarsson174-14	MK602732	MK602732
*H. pineticola* K.A. Harrison	USA	REB-94	KC571734	
*H. piperatum* Coker ex Maas Geest.	USA	REB-67	KC571720	
	USA	REB-332	JN135173	
*H. regium* K.A. Harrison	USA	SEW 93	AY569031	
*H. scleropodium* K.A. Harrison	USA	REB-352	KC571740	
	USA	REB-3	JN135186	
*H. scrobiculatum* (Fr.) P. Karst.	USA	REB-78	JN135181	
*H. spongiosipes* (Peck) Pouzar	USA	REB-52	JN135184	
	UK	RBG Kew K(M)124986	EU784269	
*H. suaveolens* (Scop.) P. Karst.	Norway	SSvantesson877	MK602736	MK602736
	Sweden	ELarsson8-14	MK602735	MK602735
*H. subsuccosum* K.A. Harrison	USA	SEW 55	AY569033	
*Sarcodon amygdaliolens* Rubio Casas	Spain	SC-2011	JN376763	
*S. aspratus* (Berk.) S. Ito			DQ448877	
			AF335110	
***S. coactus***	China	Wei 8094	MN846278	MN846287
	China	Shi 181	MN846279	MN846288
*S. fennicus* (P. Karst.) P. Karst.	Sweden	SWesterberg110909	MK602739	MK602739
	Norway	OF242833	MK602738	MK602738
*S. fuligineoviolaceus* (Kalchbr.) Pat.	Sweden	BNylen130918	MK602741	MK602741
	Norway	AMolia160201	MK602742	MK602742
*S. fuscoindicus* (K.A. Harrison) Maas Geest.	USA	OSC 113641	EU669230	EU669280
	USA	OSC 107844	EU669229	EU669279
*S. glaucopus* Maas Geest. & Nannf.	Sweden	Edvinson110926	MK602745	MK602745
	Sweden	JNitare060916	MK602744	MK602744
***S. grosselepidotus***	China	Yuan 1247	MN846273	
	China	Wei 8120	MN846274	MN846283
	China	Wei 8075	MN846276	MN846285
	China	Wei 8128	MN846277	MN846286
	China	Wei 8097	MN846275	MN846284
*S. imbricatus* (L.) P. Karst.	Norway	SSvantesson355	MK602748	MK602748
	Sweden	ELarsson384-10	MK602747	MK602747
*S. joeides* (Pass.) Bataille	Sweden	Nitare110829	MK602751	MK602751
	Sweden	KHjortstam17589	MK602750	MK602750
*S. lepidus* Maas Geest.	Sweden	JNitare110829	MK602754	MK602754
	Sweden	RGCarlsson10-065	MK602752	MK602752
*S. leucopus* (Pers.) Maas Geest. & Nannf.	Norway	OF296099	MK602755	MK602755
	Sweden	PHedberg080811	MK602757	MK602757
***S. leucopus***	China	Dai 5686	MN846282	MN846291
***S. lidongensis***	China	Wei 8365	MN846280	MN846289
	China	Wei 8329	MN846281	MN846290
*S. lundellii* Maas Geest. & Nannf.	Norway	OF295814	MK602760	MK602760
	Norway	OF242639	MK602759	MK602759
*S. martioflavus* (Snell, K.A. Harrison & H.A.C. Jacks.) Maas Geest.	Sweden	ADelin110804	MK602763	MK602763
	Norway	OF242435	MK602762	MK602762
*S. quercinofibulatus* Pérez-De-Greg., Macau & J. Carbó	Italy	JC-20090718.2	JX271818	MK602773
	USA	TENN	MG663244	
*S. scabripes* (Peck) Banker	Mexico	FCME:23240	EU293829	
	USA	REB-351	JN135191	
*S. scabrosus* (Fr.) P. Karst.	Norway	OF292320	MK602766	MK602766
	Norway	OF360777	MK602765	MK602765
*S. squamosus* (Schaeff.) P. Karst.	Norway	OF295554	MK602769	MK602769
	Norway	OF177452	MK602768	MK602768
*S. underwoodii* Banker	USA	REB-358	JN135189	
	USA	REB-119	KC571782	
*S. versipellis* (Fr.) Nikol.	Sweden	RGCarlsson11-08	MK602772	MK602772
	Sweden	RGCarlsson13-057	MK602771	MK602771
*Sarcodon* sp.		SL71	EU627610	
		TPML20130628-34	MF611700	
		SFC20140822-38	MF611702	
	Italy	OTU9	MH681180	
	New Caledonia	CY13_061	KY774274	KY774274
	China	LL_119	KX008981	
	Mexico	GO-2009-415	KC152220	
	New Zealand	PDD:105158	KP191971	KP191774

Nuclear ribosomal RNA genes were used to determine the phylogenetic position of the new species. After PCR amplification, the products were sequenced in both directions and the sequences were assembled using DNAMAN 8.0. DNA sequences were aligned with MUSCLE in MEGA7 (Kumar et al. 2016). Alignments were manually adjusted to allow maximum alignment and minimise gaps. Maximum parsimony and Bayesian analysis were applied to the ITS + LSU dataset. All characters were weighted and gaps were treated as missing data. Maximum parsimony analysis (PAUP* version 4.0b10) was used (Swofford 2002). Trees were inferred using the heuristic search option with tree bisection reconnection (TBR) branch swapping and 1,000 random sequence additions. Max-trees were set to 5000 and no-increase, branches of zero length were collapsed and all parsimonious trees were saved. Clade stability was assessed using a bootstrap (BT) analyses with 1,000 replicates (Gaget et al. 2017). Descriptive tree statistics, tree length (TL), consistency index (CI), retention index (RI), rescaled consistency index (RC) and homoplasy index (HI), were calculated for all trees generated under different optimality criteria. Maximum Likelihood (ML) analysis was performed in RAxML v8.2.4 with GTR+I+G model (Stamatakis 2014). The best tree was obtained by executing 1000 rapid bootstrap inferences and thereafter a thorough search was undertaken for the most likely tree using one distinct model/data partition with joint branch length optimisation (Stamatakis et al. 2008). Bayesian analyses with MrBayes 3.2.4 (Cannatella 2015) implementing the Markov Chain Monte Carlo (MCMC) technique and parameters predetermined with MrMODELTEST2.3 (Posada and Crandall 1998; Nylander 2004) were performed and the parameters in MrBayes were set as follows: lset nst = 6, rates = invgamma. Four simultaneous Markov chains were run starting from random trees, keeping one tree every 100^th^ generation until the average standard deviation of split frequencies was below 0.01. The value of burn-in was set to discard 25% of trees when calculating the posterior probabilities. Bayesian posterior probabilities were obtained from the 50% majority rule consensus of the trees kept. Then we used the FigTree v1.3.1 or Treev32 to visualise the resulting trees.

## Results

### Phylogenetic analyses

**Figure 1.**
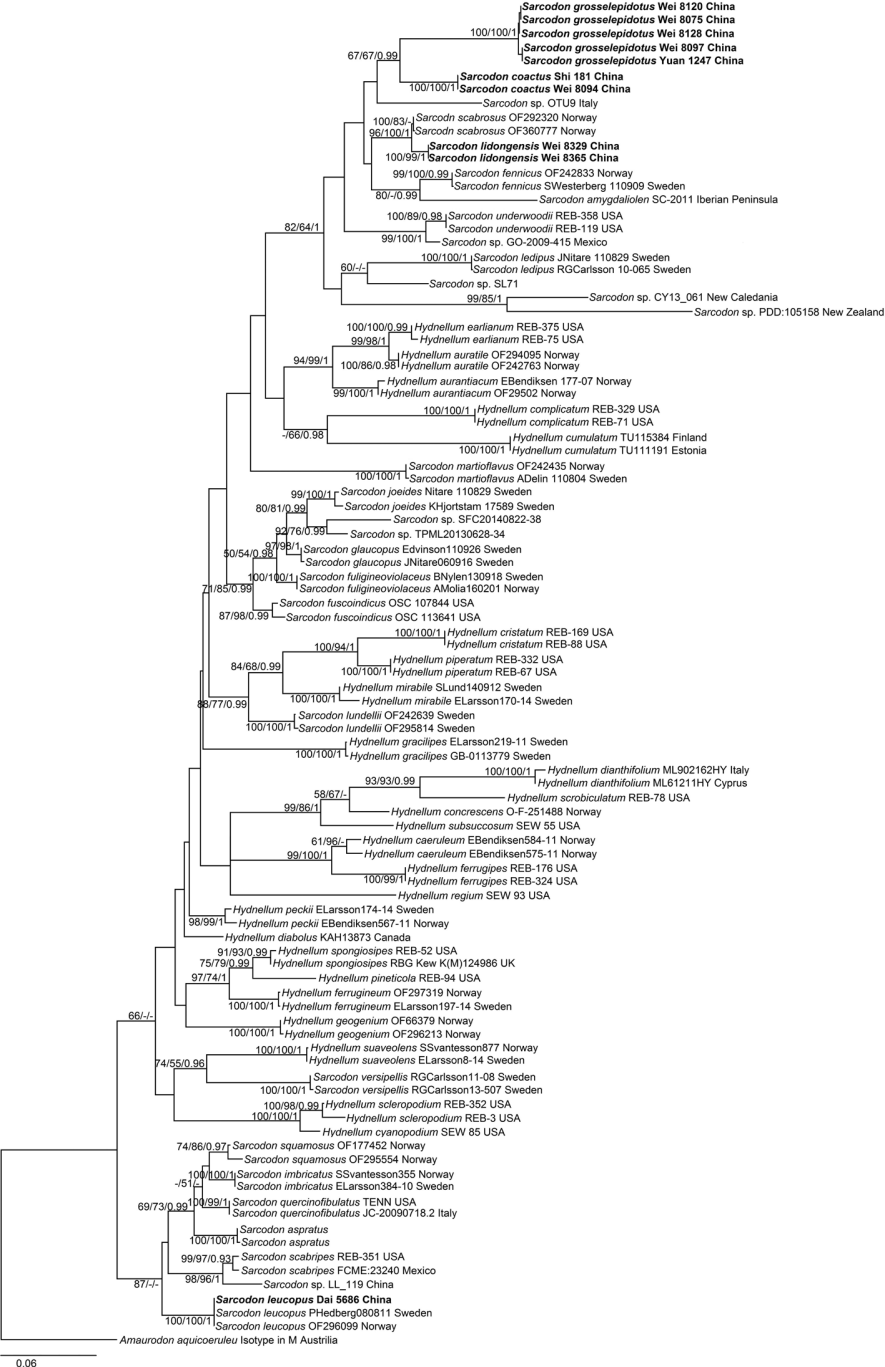
Maximum likelihood tree illustrating the phylogeny of *Sarcodon coactus*, *S. grosselepidotus*, *S. lidongensis*, *S. leucopus* and related taxa based on ITS and LSU sequence datasets. Branches are labelled with maximum likelihood bootstrap support greater than 50%, parsimony bootstrap proportions greater than 50% and Bayesian posterior probabilities greater than 0.95.

The combined ITS-LSU dataset represented 97 taxa and 1328 characters long after being trimmed. *Amaurodon aquicoerule* was used as the outgroup. The data matrix comprised 800 constant characters, 81 parsimony uninformative variable characters and 447 parsimony informative positions. Maximum parsimony analysis was performed and a strict consensus tree was obtained (TL = 2351, CI = 0.376, RI = 0.728, RC = 0.273, HI = 0.624). Bayesian analysis ran for 8 million generations and resulted in an average standard deviation of split frequencies of 0.004708. The same dataset and alignment were analysed using the ML method and a similar topology was generated. The ML tree is shown in Figure 1. In the phylogenetic tree, nine sampled specimens formed three single clades with high to full support (100% in ML, 99% or 100% in MP and 1.00 BPP) and clustered in the clade that comprised most species of *Sarcodon*. *S. coactus* and *S. grosselepidotus*, clustered together with moderate support (67% in ML, 67% in MP and 0.99 BPP). *S. lidongensis* clustered with *S. scabrosus* with strong support (96% in ML, 100% in MP and 1.00 BPP). One sampled specimen of *S. leucopus* clustered with two samples (MK602757 and MK602755) from Sweden with full support (100% in ML, 100% in MP and 1.00 BPP). It confirmed a newly recorded species of *S. leucopus* from China.

### Taxonomy

#### Sarcodon coactus

Taxon classificationFungiThelephoralesBankeraceae

Y.H. Mu & H.S. Yuan sp. nov.

F149B8A9-70B1-509D-B706-EFAB4952B890

833889

Figures 2 , 3 , 4

##### Diagnoses.

Differs from *Sarcodon thwaitesii* by slightly shorter and decurrent spines, olivaceous tissues in KOH, simple-septate hyphae in all parts of basidiocarp, narrower basidia with shorter sterigmata and smaller basidiospores.

##### Type.

**China.** Yunnan Province, Chuxiong, Zixishan Nat. Res., 24°58'28"N, 101°22'13"E, 2000 m alt., solitary to gregarious, on the ground in Fagaceae forest, 19.07.2018, *Wei 8094* (holotype: IFP 019351).

##### Etymology.

*Coactus* (Lat.), refers to the felted pileal surface.

##### Description.

Basidiocarps annual, solitary to gregarious, soft and fleshy when fresh, becoming firm and light in weight upon drying; taste none, odour farinaceous when dry. Pileus planar, ellipsoid when young, later round with age, up to 35 mm across and 4–8 mm thick at centre. Pileal surface reddish-brown (8D5) to dark brown (8F8), azonate, pubescent, floccose to felted when fresh, becoming smooth, rugose, scrobiculate when dry; margin white (7A1) when fresh, greyish-brown (7D3) with age, incurved, rarely lobed. Spine surface white (4A1) to yellowish-white (4A2) when fresh, brownish-orange (5C5) to yellowish-brown (5F6) when dry; spines up to 2.1 mm long, base up to 0.3 mm diam., conical, 3–5 per mm, decurrent on stipe, without spines at pileus margin, brittle when dry. Context not duplex, up to 6 mm thick, light brown (5D5), firm; Stipe central, up to 5.5 cm long and 1.3 cm diam., fleshy, greyish-brown (8D3) to violet brown (10F7) when fresh, becoming hollow with age, greyish-orange (5B3) to dark brown (7F7) upon drying, rugous, columniform or attenuate below with bulbous base when old.

Hyphal structure. Hyphal system monomitic; generative hyphae with simple-septa, CB–, IKI–; tissues olivaceous in KOH.

Context. Generative hyphae hyaline, thin-walled, rarely branched, simple-septate, inflated, partly short-celled, interwoven, mostly 4–10 μm diam.

Spines. Tramal hyphae hyaline, thin-walled, frequently branched, more or less parallel along spines, frequently simple-septate, straight, 2–5 μm diam. Cystidia and cystidioles absent. Basidia clavate, thin-walled, with four sterigmata (3.1–5.2 μm long), simple-septate at base, 16.5–50 × 6.2–9.4 μm; basidioles similar to basidia.

Basidiospores irregular subglobose, brown, thin-walled, tuberculate, CB–, IKI–, (5.1–)5.7–7(–7.1) × (4.6–)4.7–5.9(–6) μm, Lm = 6.2 μm, Wm = 5.3 μm, Q = 1.17–1.18 (n = 60/2); tuberculi usually isolated or grouped in 2 or more, bi- to trifurcate-like in shape, up to 1.0 μm long.

##### Additional specimen examined

– **China.** Yunnan Province, Maguan County, On the way from Dalishu Township to Damagu Village, 23°4'55"N, 104°12'59"E, 1616 m alt., solitary, on the ground in Fagaceae forest, 7.08.2017, *Shi 181* (IFP 019352).

**Figure 2.**
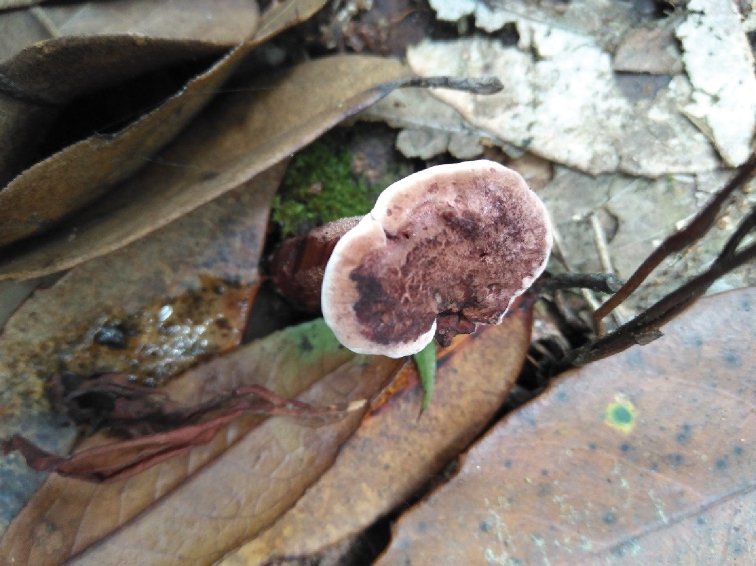
A basidiocarp of *Sarcodon coactus* (holotype: IFP 019351).

**Figure 3.**
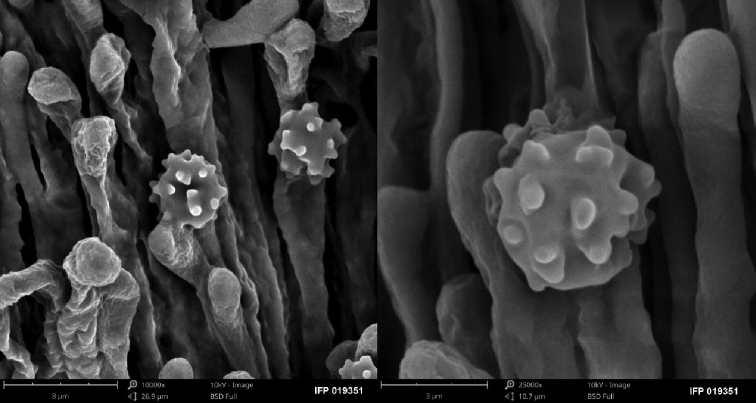
SEM of basidiospores of *Sarcodon coactus* (holotype: IFP 019351).

**Figure 4.**
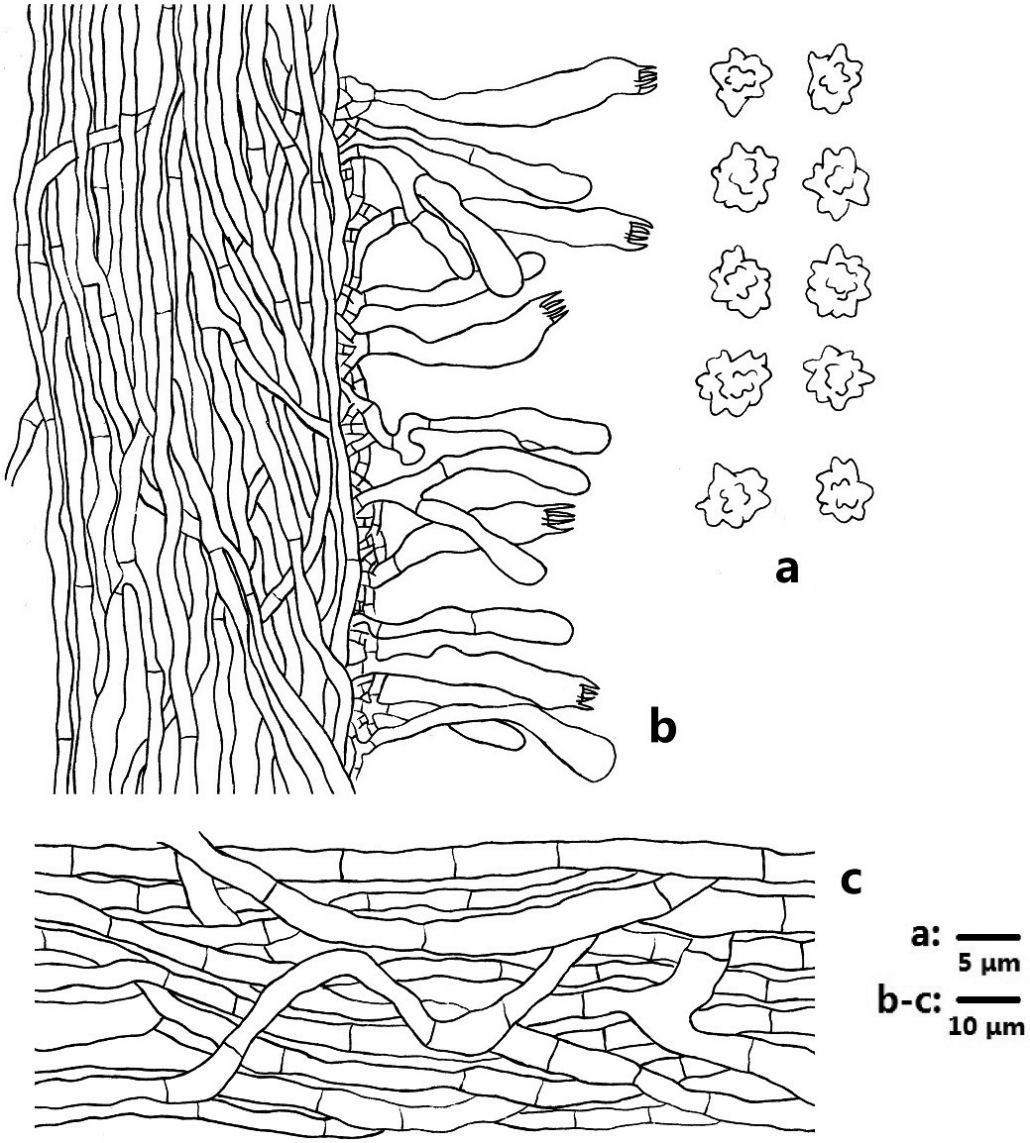
Microscopic structures of *Sarcodon coactus* (drawn from IFP 019351) **a** basidiospores **b** section of hymenophoral trama with basidia **c** hyphae from pileal context.

#### Sarcodon grosselepidotus

Taxon classificationFungiThelephoralesBankeraceae

Y.H. Mu & H.S. Yuan sp. nov.

E7E671B0-C24A-580C-B65E-65D45C21D111

833890

Figures 5 , 6 , 7

##### Diagnoses.

Differs from *Sarcodon lepidus* in having shorter and slightly wider spines, fragrant odour, narrower hyphae in context, slightly wider basidia with shorter sterigmata and wider basidiospores.

##### Type.

**China.** Yunnan Province, Chuxiong, Zixishan Nat. Res., 24°58'28"N, 101°22'13"E, 2000 m alt., solitary or gregarious, on the ground in Fagaceae forest, 1.08.2005, *Yuan 1247* (holotype: IFP 012529).

##### Etymology.

*Grosselepidotus* (Lat.), from the Latin word grosse and lepidotus, in reference to the coarsely scaled pileal surface.

##### Description.

Basidiocarps annual, solitary to gregarious, soft and freshy when fresh, becoming fragile and light in weight upon drying; taste none, odour mildly fragrant when dry. Pileus infundibuliform or circular when young, later planar and ellipsoid to round with age, occasionally deeply fissured, up to 75 mm diam. and 4–8 mm thick at centre. Pileal surface pale orange (6A3) to dark ruby (12F8), azonate, glabrous with ascending, broad and dark brown (9F5) scales when fresh, becoming scabrous, rugose when dry; margin inflexed and wavy, sometimes lobed with age. Spine surface white (4A1) to pale yellow (4A3) when fresh, light brown (6D6) to dark brown (6F8) when dry; spines up to 1.4 mm long, base up to 0.3 mm diam., conical, 4–6 per mm, strongly decurrent on stipe, without spines at pileus margin, brittle when dry. Context not duplex, up to 5 mm thick, greyish-orange (5B5), firm; Stipe central to lateral, up to 9.5 cm long and 2 cm diam., fleshy when fresh, firm upon drying, brownish-yellow (5C7) to dark brown (7F7), creased, inside solid, cylindrical or attenuate below with bulbous base when old.

Hyphal structure. Hyphal system monomitic; generative hyphae with simple-septa, CB–, IKI–; tissues olivaceous in KOH.

Context. Generative hyphae hyaline, thin-walled, rarely branched, simple-septate, inflated, interwoven, mostly 7–11 μm diam.

Spines. Tramal hyphae hyaline, thin-walled, occasionally branched, more or less parallel along spines, frequently simple-septate, straight, 2–5 μm diam. Cystidia and cystidioles absent. Basidia clavate, thin-walled, with four sterigmata (2.5–5 μm long), simple-septate at base, 23.5–55.5 × 5.3–8.2 μm; basidioles similar to basidia.

Basidiospores irregular ellipsoid to globose, brown, thin-walled, tuberculate, CB–, IKI–, (5–)5.1–6.4(–6.6) × (4–)4.1–5.9(–6) μm, Lm = 5.5 μm, Wm = 4.9 μm, Q = 1.13–1.19 (n = 60/2); tuberculi usually isolated, sometimes grouped in 2 or more, bi- to trifurcate-like in shape, up to 0.7 μm long.

##### Additional specimen examined

– **China.** Yunnan Province, Chuxiong, Zixishan Nat. Res., 24°58'28"N, 101°22'13"E, 2000 m alt., solitary to gregarious, on the ground in Fagaceae forest, 19.07.2018, *Wei 8075* (IFP 019353), *Wei 8097* (IFP 019354), *Wei 8120* (IFP 019355) and *Wei 8128* (IFP 019356).

**Figure 5.**
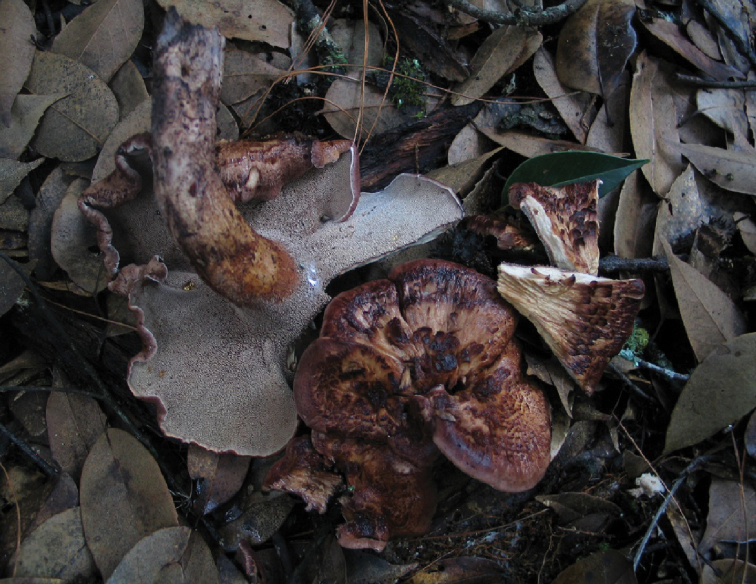
Basidiocarps of *Sarcodon grosselepidotus* (holotype: IFP 012529).

**Figure 6.**
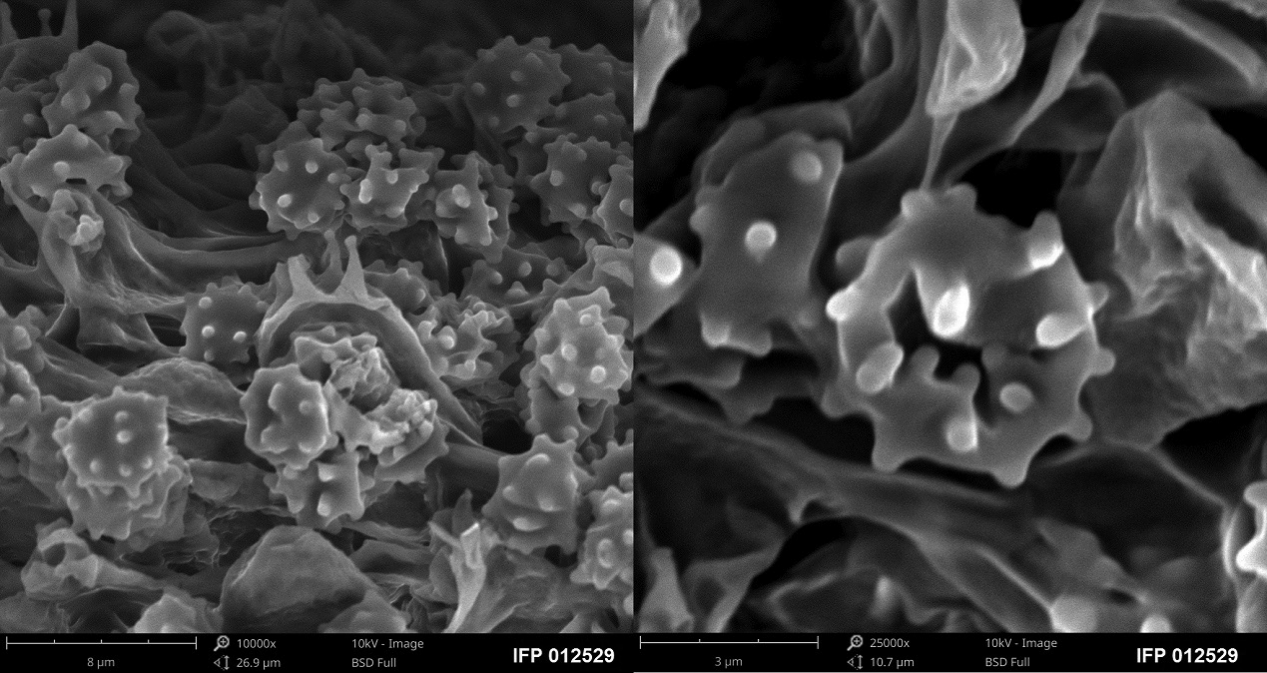
SEM of basidiospores of *Sarcodon grosselepidotus* (holotype: IFP 012529).

**Figure 7.**
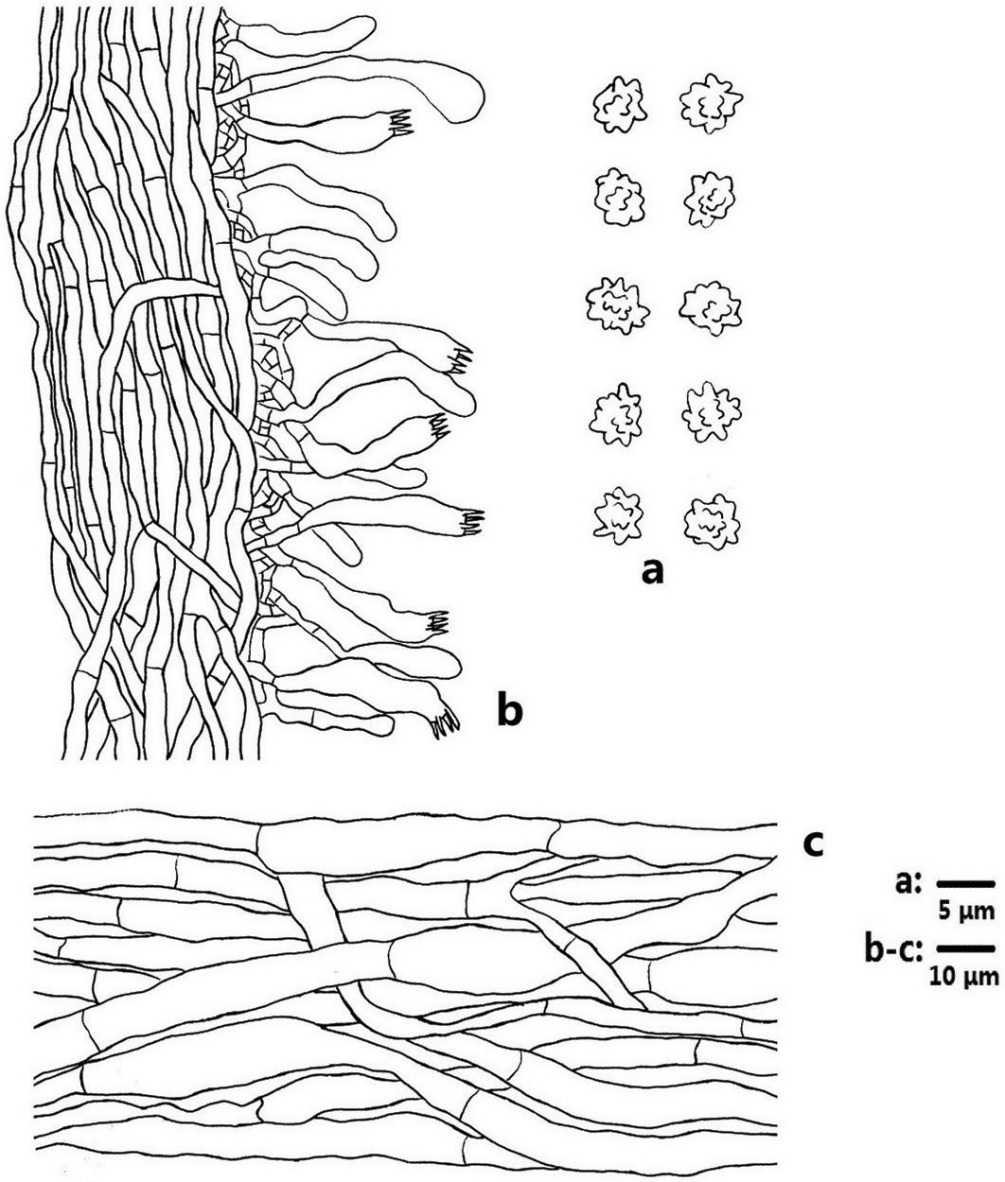
Microscopic structures of *Sarcodon grosselepidotus* (drawn from IFP 012529) **a** basidiospores **b** section of hymenophoral trama with basidia **c** hyphae from pileal context.

#### Sarcodon lidongensis

Taxon classificationFungiThelephoralesBankeraceae

Y.H. Mu & H.S. Yuan sp. nov.

027C929B-90BB-5BA4-B092-990294BEAB24

833891

Figures 8 , 9 , 10

##### Diagnoses.

Differs from *Sarcodon joeides* in having shorter, more or less decurrent spines, the absence of gloeoplerous hyphae, shorter basidia sterigmata and narrower basidiospores.

##### Type.

**China.** Yunnan Province, Lidong County, Qunlong Villa, 26°35'28"N, 99°24'16"E, 2400 m alt., solitary to concrescent, on the ground in Fagaceae forest, 24.07.2018, *Wei 8365* (holotype: IFP 019357).

##### Etymology.

*Lidongensis*, refers to Lidong County, where the specimens were collected.

##### Description.

Basidiocarps annual, simple to concrescent, soft and freshy when fresh, becoming firm and light in weight upon drying; taste bitterish, odour farinaceous when dry. Pileus planar and circular when young, later plano-convex to somewhat depressed and regular orbicular with age, up to 35 mm across and 5–8 mm thick at centre. Pileal surface light brown (6D7) to brown (7E8), azonate, velutinate, then matted, appressed squamose to rimose when fresh, and purplish-brown at the pileal margin, dark brown in centre, becoming scrobiculate and verrucose when dry; margin incurved and occasionally incised with age. Spine surface greyish-orange (6B3) to brown (6E6) when fresh, light brown (6D5) to brown (6E7) when dry; spines up to 1 mm long, base up to 0.2 mm diam., conical, 4–6 per mm, more or less decurrent on stipe, with spines at pileus margin, brittle when dry. Context not duplex, up to 6 mm thick, orange white (5A2) to yellowish-brown (5D6), firm; stipe central, up to 4.5 cm long and 1 cm diam., fleshy when fresh, rigid upon drying, light brown (6D6) to dark brown (6F6), fibrillose, inside solid, cylindrical or broadened below with bulbous base when old.

Hyphal structure. Hyphal system monomitic; generative hyphae with simple-septa, CB–, IKI–; tissues olivaceous in KOH.

Context. Generative hyphae hyaline, thin-walled, occasionally branched, simple-septate, inflated, interwoven, mostly 5–9 μm diam.

Spines. Tramal hyphae hyaline, thin-walled, occasionally branched, more or less parallel along spines, frequently simple-septate, straight, sometimes flexuous and collapsed, 2–4 μm diam. Cystidia and cystidioles absent. Basidia clavate, thin-walled, with four sterigmata (2.0–3.0 μm long), simple-septate at base, 19.2–39.3 × 3.0–7.2 μm; basidioles similar to basidia.

Basidiospores irregular ellipsoid to subglobose, brown, thin-walled, tuberculate, CB–, IKI–, (4–)4.1–6(–6.1) × (3.9–)4–5(–5.1) μm, Lm = 5.5 μm, Wm = 4.9 μm, Q = 1.15–1.20 (n = 60/2); tuberculi usually isolated or grouped in 2 or more, bi- to trifurcate-like in shape, up to 1.0 μm long.

##### Additional specimen examined

– **China.** Yunnan Province, Lidong County, Qunlong Villa, 26°35'28"N, 99°24'16"E, 2400 m alt., solitary to concrescent, on the ground in Fagaceae forest, 24.07.2018, *Wei 8329* (IFP 019358).

**Figure 8.**
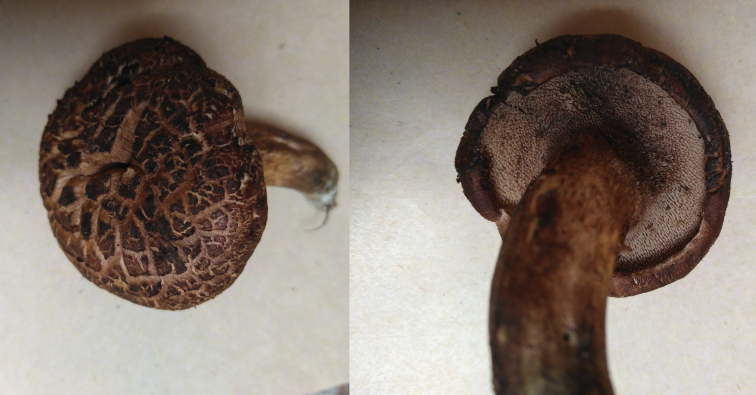
Basidiocarps of *Sarcodon lidongensis* (holotype: IFP 019357).

**Figure 9.**
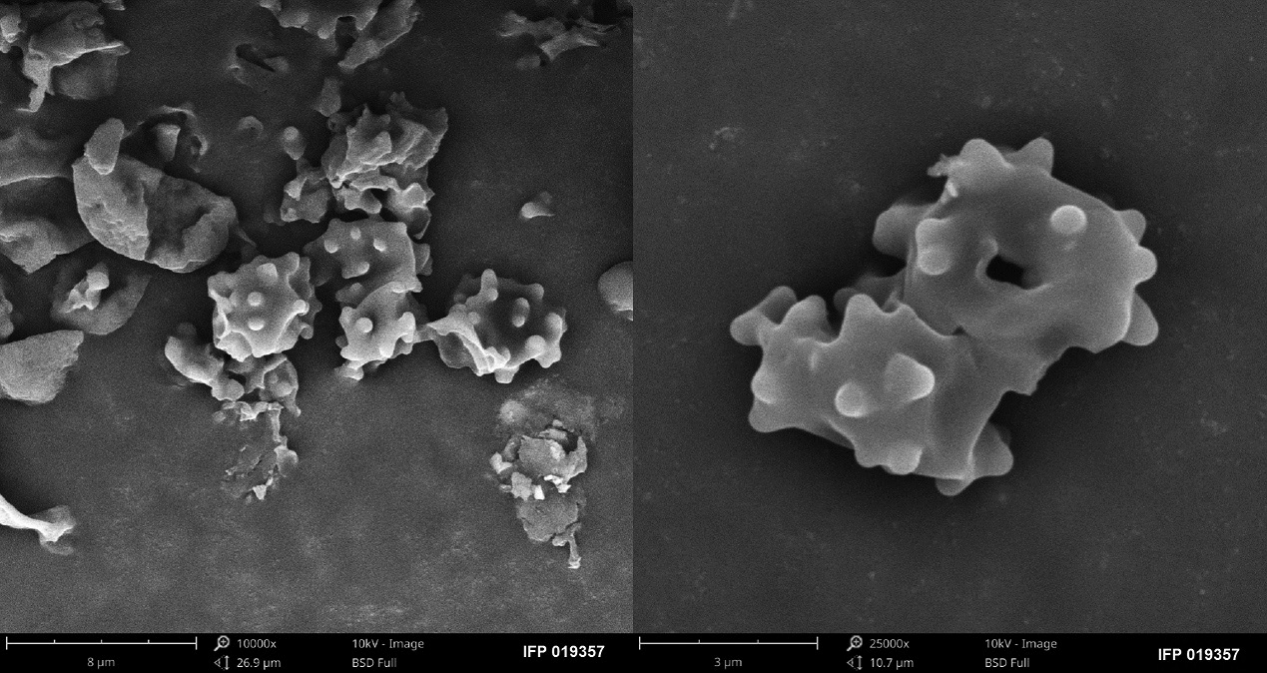
SEM of basidiospores of *Sarcodon lidongensis* (holotype: IFP 019357).

**Figure 10.**
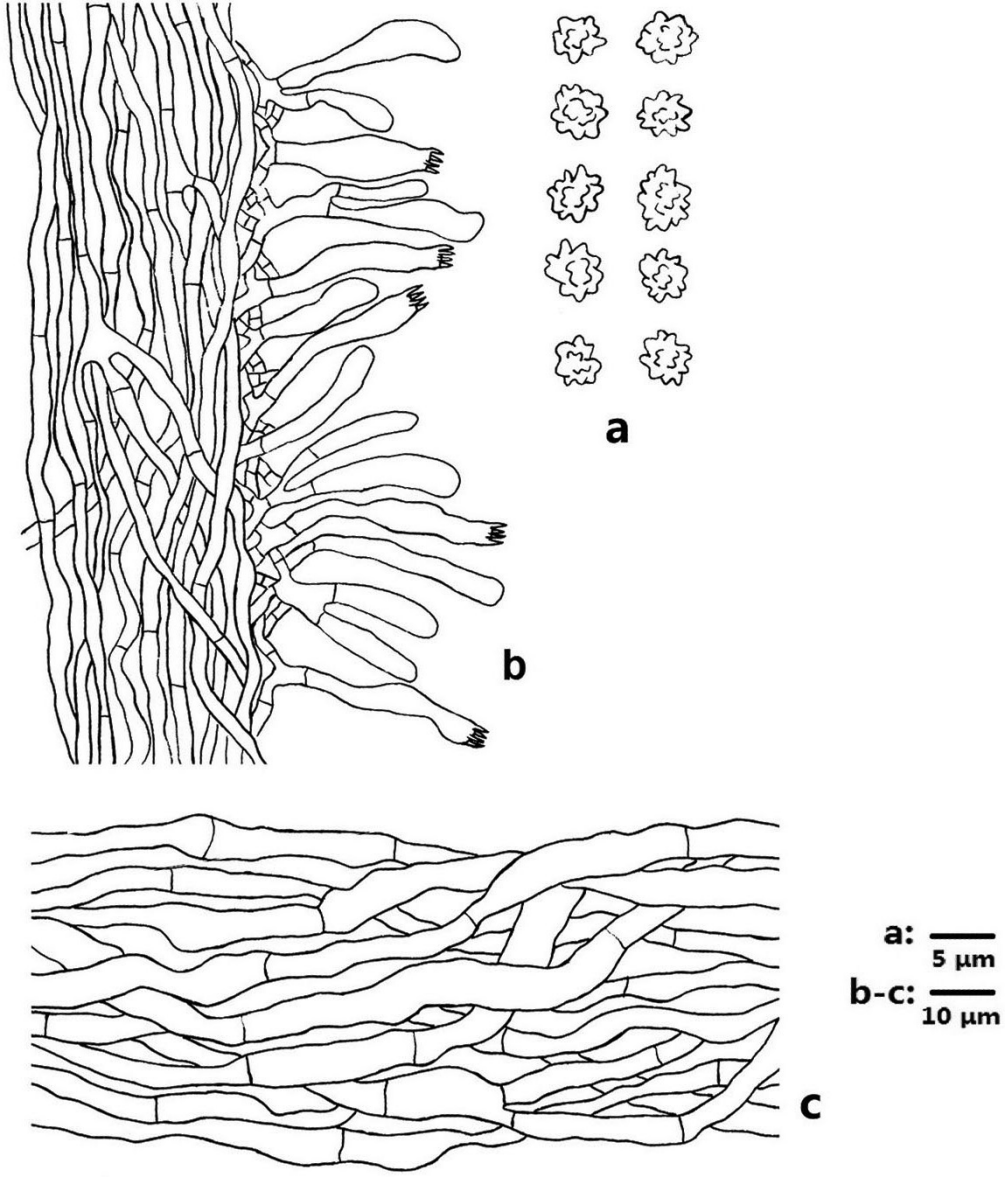
Microscopic structures of *Sarcodon lidongensis* (drawn from IFP 019357) **a** basidiospores **b** section of hymenophoral trama with basidia **c** hyphae from pileal context.

#### Sarcodon leucopus

Taxon classificationFungiThelephoralesBankeraceae

(Pers.) Maas Geest. & Nannf., Svensk Botanisk Tidskrift 63: 415, 1969.

D881D307-071C-5DC1-94E4-F39136E0A015

##### Diagnoses.

Morphological and nuc ITS rDNA + nuc LSU rDNA sequences analyses confirmed the new record species, which is described in detail by Mleczko et al. (2011). This species was recorded by several European countries, such as Estonia, Finland, Bulgaria and Sweden and was frequently placed on the Red List (Rassi et al. 2001; Gärdenfors 2005; Gyosheva et al. 2006; Parmasto 2009).

##### Specimen examined

– **China.** Xizang Auto. Reg., Linzhi, Bayi Town, 92°09'14"E, 26°52'26"N, 3000 m alt., solitary or gregarious, on the ground of alpine *Pinus* forest, 3.08.2004, *Dai 5686* (IFP 010196).

## Discussion

Three new species of *Sarcodon* were described, based on the morphological characteristics and molecular data and were the first new species described from China. Phylogenetic analyses of the nuc ITS rDNA + nuc LSU rDNA dataset by ML, MP and Bayes in this study showed a low level of support in the deeper nodes of the topology, but high support at the species level. The result is in keeping with previous reports (Baird et al. 2013; Larsson et al. 2019).

The felted pileal surface is the main feature of *Sarcodon coactus* and this is consistent with *S. repandus* and *S. thwaitesii*. However, *S. repandus* differs from *S. coactus* by a larger pileus (up to 50 mm vs. 35 mm in *S. coactus*) with longer spines (up to 4 mm vs. 2.1 mm in *S. coactus*), clamped hyphae and wider hyphae in the context (up to 25 µm) (Maas Geesteranus 1971). *S. thwaitesii* resembles *S. coactus* in having an azonate pileal surface, central and hollow stipe when old and thin-walled hyphae in trama. However, *S. thwaitesii* differs from *S. coactus* by slightly longer (up to 3 mm vs. 2.1 mm in *S. coactus*) and not decurrent spines, blue-green tissues in KOH, clamped hyphae in all parts of the basidiocarp, wider basidia (10–11 µm vs. 6.2–9 μm in *S. coactus*) with longer sterigmata (5.4–9 µm vs. 3.1–5.2 μm in *S. coactus*) and larger basidiospores (8.1–9.4 × 5.8–7.2 µm vs. 5.7–7 × 4.7–5.9 μm in *S. coactus*) (Maas Geesteranus 1971).

*Sarcodon grosselepidotus* presents a distinct characteristic: pileal surface with ascending and coarse scales, that coincide with that of *S. imbricatus* and *S. lepidus* (Maas Geesteranus 1975; Baird et al. 2013). However, *S. imbricatus* is differentiated from the new species by having longer spines (up to 8 mm vs. 1.4 mm in *S. grosselepidotus*), clamped hyphae in all parts of the basidiocarp, presence of gloeoplerous-like hyphae and larger basidiospores (8–9 × 7–8 μm vs. 5.1–6.4 × 4.1–5.9 μm in *S. grosselepidotus*) (Maas Geesteranus 1971; Baird et al. 2013). *S. lepidus* differs from *S. grosselepidotus* by having longer spines (up to 3 mm vs. 1.4 mm in *S. grosselepidotus*), farinaceous odour, wider hyphae in the context and narrower basidiospores (3.6–4.3 µm vs. 4.1–5.9 μm in *S. grosselepidotus*) (Maas Geesteranus 1975).

*Sarcodon coactus* and *S. grosselepidotus* are closely related in the phylogenetic tree and share similar morphological and anatomical characteristics: solitary to gregarious basidiocarps with round pileus, central and columniform stipe, decurrent spines, context tissue becoming olivaceous in KOH and isolated or grouped tuberculi. However, *S. grosselepidotus* can be differentiated by infundibuliform basidiocarps, fissured pileus, coarse and scaly pileal surface, shorter spines (up to 1.4 mm vs. 2.1 mm in *S. coactus*) and slightly shorter tuberculi (up to 0.7 μm vs. 1 μm in *S. coactus*).

*Sarcodon lidongensis* and *S. scabrosus* reveal a close phylogenetic relationship according to the phylogenetic tree. In morphology, *S. lidongensis* is similar to *S. scabrosus* in having a single or gregarious basidiocarp with convex to planar or depressed pileus, brown and scaled pileal surface, central and terete stipe, olivaceous tissues in KOH and basidiospores of similar shape. However, *S. scabrosus* is differentiated by a larger pileus (up to 15 cm across) with longer spines (up to 8 mm vs. 1 mm in *S. lidongensis*), wider basidia (7–9 μm vs. 3.0–7.2 μm in *S. lidongensis*) with longer sterigmata (4–5 μm vs. 2–3 μm in *S. lidongensis*) and larger basidiospores (6–7 × 5–7 μm vs. 4.1–6 × 4–5 μm in *S. lidongensis*) (Maas Geesteranus 1971; Baird 1986; Baird et al. 2013).

*Sarcodon joeides* is similar to *S. lidongensis* in having simple basidiocarps with plano-convex or depressed pileus, mottling or tear-like pileal surface, appressed scales, central and terete stipe, olivaceous tissue in KOH, inflated and interwoven hyphae in the context and tuberculate basidiospores of similar shape. However, it differs from *S. lidongensis* in having longer, decurrent to strongly decurrent spines (up to 3 mm vs. 1 mm in *S. lidongensis*), presence of gloeoplerous-like hyphae, longer basidia sterigmata (4–5 μm vs. 2–3 μm in *S. lidongensis*) and wider basidiospores (5–6 μm vs. 4–5 μm in *S. lidongensis*) (Baird et al. 2013).

The specimens, involved in this study, were collected from the forests dominated by Fagaceae trees such as *Quercus acutissima*, *Lithocarpus dealbatus*, *Castaopsis orthacantha* and a small portion of coniferous trees, for instance, *Pinus armandii*. We speculated that these species may form an ectomycorrhizal association with Fagaceae trees. The new record sample was fully identical with *S. leucopus* described by Mleczko et al. (2011) in morphology and molecular analysis and pine and spruce were primary ectomycorrhizal companions of this fungus.

### Key to species of *Sarcodon* from China

**Table:** 

1	Basidiospores lengths in the range 8–10 μm, hyphae with frequent clamp connections in all parts of basidiocarps	***S. leucopus***
–	Basidiospores lengths in the range 4–7 μm, hyphae without clamp connection in any part of basidiocarps	**2**
2	Pileal surface not scaled, felted when fresh, spines up to 2.1 mm	***S. coactus***
–	Pileal surface scaled when fresh, spines up to 1.4 mm	**3**
3	Basidiocarps of occasionally deeply fissured pileus, pileal surface with ascending squama	***S. grosselepidotus***
–	Basidiocarps of not deeply fissured pileus, pileal surface with appressed squama	***S. lidongensis***

## Supplementary Material

XML Treatment for Sarcodon coactus

XML Treatment for Sarcodon grosselepidotus

XML Treatment for Sarcodon lidongensis

XML Treatment for Sarcodon leucopus
